# Prevalence of elective cosmetic surgery and its relationship with socioeconomic and mental health: A cross‐sectional study in west of Iran

**DOI:** 10.1002/hsr2.872

**Published:** 2022-10-17

**Authors:** Ahoora Ghorbani, Bakhtiar Piroozi, Hossein Safari, Azad Shokri, Abbas Aqaei, Fayegh Yousefi, Maziar Nikouei, Mahdi Rafieemovahhed

**Affiliations:** ^1^ Research Student Committee Kurdistan University of Medical Sciences Sanandaj Iran; ^2^ Social Determinants of Health Research Center, Research Institute for Health Development Kurdistan University of Medical Sciences Sanandaj Iran; ^3^ Health Promotion Research Center Iran University of Medical Sciences Tehran Iran; ^4^ School of Nursing and Midwifery Qazvin University of Medical Sciences Qazvin Iran

**Keywords:** cosmetic surgery, Iran, mental health, spiritual health

## Abstract

**Background and Aims:**

The aim of this study was to investigate the prevalence and desire towards performing elective cosmetic surgery and its relationship with socioeconomic, mental, and spiritual health in Sanandaj, the capital of Kurdistan province in the west of Iran.

**Methods:**

This cross‐sectional descriptive‐analytical study was performed on 492 subjects in Sanandaj using multistage sampling method. Data collection tools included two checklists and two questionnaires. Data analysis was performed using the Chi‐square test and multiple logistics regression model by the help of SPSS software version 20.

**Results:**

The mean (standard deviation) age of participants was 32.58 (9.67) years. The prevalence of cosmetic surgery and the desire to perform it were 12.8% (*n* = 63) and 19.1% (*n* = 94), respectively. The most common type of cosmetic surgery was rhinoplasty with 5.5% (*n* = 27). The prevalence of symptoms of mental disorders among people with cosmetic surgery and people willing to perform cosmetic surgery was significantly higher than all subjects (*p* < 0.001). Female gender (odds ratio [OR] = 1.98; 95% confidence interval [CI]: 1.06–3.68) and having mild (OR = 3.01 95% CI: 1.06–3.68) and moderate to severe symptoms of mental disorder (OR = 7.59; 95% CI: 3.90–14.75) were among the influential variables on performing cosmetic surgery.

**Conclusion:**

Both the prevalence and desire towards performing cosmetic surgery are high in Sanandaj and this needs the attention of health policy makers. Designing targeted interventions with an emphasis on the findings of this study is proposed to reduce these practices.

## 1 INTRODUCTION

Cosmetic surgery is defined as an optional invasive procedure to change and correct the shape of the body in the absence of a specific disease, injury, or birth defect.^1^ A common defense of cosmetic procedures is to help improve self‐confidence and thus allow the patient to live a happier life.^2^ Statistics show that the number of cosmetic surgeries in the world is increasing every year compared to the previous year.^3^ Breast augmentation, liposuction, blepharoplasty, rhinoplasty, abdominoplasty, and breast and face lift and breast reduction are the most common cosmetic surgeries in the world.^4^ The United States has the largest number of cosmetic surgeries in the world, with more than 2.3 million cosmetic surgeries registered in the country alone in 2020.^5^ After the United States, the highest number of cosmetic surgeries are respectively performed in Brazil, Mexico, Germany, India, Japan, Argentina, Russia, Italy, Colombia, South Korea, and Turkey.^6^

Cosmetic surgery has become a social value despite its risks and side effects. The increasing importance of appearance in the contemporary world, the effect of fashion, exposure to the media, the spread of social networks, social pressure, encouragement of doctors, advertising, and the cost‐effectiveness of cosmetic surgery are among the factors increasing the rate of these operations in the world.^7^, ^8^, ^9^, ^10^ Based on the findings of some studies, there is a significant relationship between cosmetic surgery and personality disorders, body dysmorphic disorder, obsessive‐compulsive disorder, depression, anxiety, and low self‐esteem.^7^, ^11^, ^12^, ^13^, ^14^, ^15^, ^16^

The desire to perform cosmetic surgeries has social, psychological, and cultural roots.^17^, ^18^ Cosmetic surgery has its own consequences. In addition to the medical consequences, the economic burden of cosmetic surgery is also a matter of concern. Given that cosmetic surgery can impose risks and physical and psychological complications and a high economic burden on individuals, households, and health systems; therefore, health policymakers and managers must make decisions, design interventions, and evidence‐based policies to try to control the unbridled increase of this type of surgery in the community.^19^, ^20^, ^21^, ^22^, ^23^ In this regard, in some countries, preoperative psychological assessments are required to diagnose patients who are unsuitable for cosmetic surgery.^24^

Although exact statistics on the prevalence of cosmetic surgery in Iran are not available, reports indicate that Iran is one of the top countries in the world in terms of popularity of cosmetic surgery the demand for these surgeries is increasing and these changes have provided a suitable market for plastic surgeons.^7^, ^25^ According to unofficial sources, Iran ranks first in the rate of rhinoplasty worldwide.^26^ A study conducted in Iran in 2011 reported a 15% prevalence of cosmetic surgery among women.^27^

According to the literature, there are very few studies in Iran on the prevalence of cosmetic surgery and the factors influencing the desire towards performing this type of surgery, and there is a deep knowledge gap to provide evidence‐based policy in this area. Also, no study has been done in this regard in Kurdistan province. The aim of this study was to investigate the prevalence of cosmetic surgery and the desire to perform it as well as its relationship with mental and spiritual health in Sanandaj, the capital of Kurdistan province in the west of Iran.

## 2 MATERIALS AND METHODS

This cross‐sectional descriptive‐analytic study was performed on subjects aged 18–49 in Sanandaj, the capital of Kurdistan province in the west of Iran, with a population of 450,000 in 2021.

### 2.1 Sample size and sampling method

The sample size was determined 493 individuals using the following formula and taking into account *p* = 0.27 (prevalence of desire to perform cosmetic surgery),^28^ *d* = 0.04 (accuracy rate), and significance level of 95% (Equation 1).

(1) $$n=\frac{Z_{1-\frac{\alpha }{2}\times P(1-P)}^{2}}{d^{2}}=493.$$

Sampling was performed in several steps. First, among the comprehensive urban health centers of Sanandaj, 15 centers were randomly selected, and among the households covered by each center, 33 households were systematically selected. There was a list of households, their address, and contact number in comprehensive health centers. Then, by referring to the door of the households, questionnaires and checklists were completed by trained interviewers based on self‐report for the first person above 18–49 years who was willing and able to participate in the study. Participation in the study was voluntary and written consent was obtained from the individuals. The list, home address, and contact number of the covered households were obtained from the selected centers. In this study, the inclusion criteria were to be between 18 and 49 years old and willing to participate in the study, and the exclusion criteria were to have a mental disability or any disability such that the person is unable to understand and answer the questions in the questionnaire.

### 2.2 Data collection tools

Data collection tools included two researcher‐made checklists and two standard questionnaires.

Checklist 1 covered demographic information of individuals including gender, age, level of education, place of residence, employment and income status.

Checklist 2 included a history of cosmetic surgery and a desire to perform it. Having a history of cosmetic surgery was assessed using the following questions:

*Have you ever had cosmetic surgery? Yes □ No □*

*If the answer is “yes”, the type of cosmetic surgery should be mentioned?*

*Abdominoplasty□ Rhinoplasty□ Liposuction□ Blepharoplasty□ Breast□ Facelift□ Cheek Implant□ Otoplasty□ Hair Transplantation□ Other□*

The willingness to perform cosmetic surgery was also assessed using the following questions:

*Do you want to have cosmetic surgery on one of your body parts? Yes □ No □*

*If the answer is “yes”, the type of cosmetic surgery should be mentioned?*

*Abdominoplasty□ Rhinoplasty□ Liposuction□ Blepharoplasty□ Breast□ Facelift□ Cheek Implant□ Otoplasty□ Hair Transplantation□ Other□*

Questionnaire No. 1 was the General Health Questionnaire‐28.^29^ This questionnaire is a general tool for measuring general health. The 28‐item General Health Questionnaire was developed by Goldberg and Hiller (1979) and has four subscales, each with 7 questions. The mentioned subscales include somatic, anxiety/insomnia, social dysfunction, and severe depression symptoms. The validity and reliability of the Persian version of this questionnaire have been tested and confirmed in previous studies. The reliability of the questionnaire was reported to be 90% using Cronbach's alpha.^30^, ^31^

The Likert scoring method is used in the general health questionnaire. The total score of the four subscales ranges from 0 to 84; the cutoff point for the test is considered to be 23. In other words, individuals whose score is ≥23 are suspected of having reduced mental health and those whose score is <23 are considered healthy. Reduced mental health status is categorized as follows: a score of 23–40 indicates mild symptoms, 41–60 indicates moderate symptoms and 61–80 indicates severe symptoms. For each subscale of mental health, a score of 6 was considered as a cutoff point. Scores ≥6 indicate a suspected disorder and scores <6 indicate good health. In each of the subscale of mental health, mean scores of 7–11, 12–16, and 17–21 indicate symptoms of mild, moderate, and severe illness, respectively.^32^

Questionnaire No. 2 was the spiritual well‐being scale of Paloutzian and Ellison.^33^ This questionnaire consists of 20 items and two subscales, so 10 items measure religious health and the other 10 items measure existential health. The spiritual health score is the sum of these two subgroups, the range of which is between 20 and 120. The answer to the phrases is graded in a 6‐option Likert scale. In this questionnaire, spiritual health is divided into three levels: low (20–40), medium (41–99), and high (100–120). The validity and reliability of the Persian version of this questionnaire have been tested and confirmed in previous studies. The reliability of the questionnaire was reported to be 82% using Cronbach's alpha.^34^

### 2.3 Data analysis

Descriptive statistical tests including frequency, percentage, mean, and standard deviation were used to describe the data. Also, Chi‐square test was used to examine the relationship between demographic and socioeconomic variables, mental and spiritual health with a history of cosmetic surgery (yes/no), and inclination for performing cosmetic surgery (yes/no). The variables in Tables 1 and 3 with a *p*‐value < 0.2 based on univariate analysis were entered into the multiple logistic regression model and the odds ratio and confidence interval were calculated for them. SPSS software version 20 was used to analyze the data

**Table 1:** History of cosmetic surgery and desire to perform it based on the studied variables

Variable	Number (%)	Have cosmetic surgery		Desire to perform cosmetic surgery	
		Number (%)	*p*‐value	Number (%)	*p*‐value
Sex					
Female	279 (56.6)	46 (16.5)	0.005	61 (21.9)	0.07
Male	214 (43.4)	17 (7.9)		33 (15.4)	
Education					
Illiterate and elementary	76 (15.4)	8 (10.5)		9 (11.8)	
Middle school	120 (24.3)	14 (11.7)	0.413	25 (20.8)	0.329
High school	173 (35.1)	28 (16.2)		37 (21.4)	
Academic	124 (25.2)	13 (10.5)		23 (18.5)	
Marital status					
Single	245 (49.7)	32 (13.1)	0.852	54 (22.0)	0.095
Married	248 (50.3)	31 (12.5)		40 (16.1)	
Employment status					
Employed	233 (47.3)	29 (12.4)		40 (17.2)	
Unemployed	161 (32.7)	19 (11.8)	0.718	26 (16.1)	0.032
Housekeeper	99 (20.1)	15 (15.2)		28 (28.3)	
Household's income					
5 Million Toman>	259 (52.5)	33 (12.7)		53 (20.5)	
5–10 Million Toman	171 (34.7)	26 (15.2)	0.195	33 (19.3)	0.370
15 Million Toman<	63 (12.8)	4 (6.3)		8 (12.7)	
Total	**493 (100)**	**63 (12.8)**	**‐**	**94 (19.1)**	**‐**

**Table 2:** Prevalence of cosmetic surgery and the desire to perform it by gender and type of operation

Variable	Female		Male		Total	
	Have	Desire	Have	Desire	Have	Desire
Rhinoplasty	20 (7.2)	22 (7.9)	7 (3.3)	12 (5.6)	27 (5.5)	34 (6.9)
Liposuction	3 (1.1)	11 (3.9)	1 (0.5)	4 (1.9)	4 (0.8)	15 (3.0)
Abdominoplasty	5 (1.8)	10 (3.6)	1 (0.5)	4 (1.9)	6 (1.2)	14 (2.8)
Blepharoplasty	5 (1.8)	6 (2.2)	0	0	5 (1.0)	6 (1.2)
Breast	6 (2.2)	11 (3.9)	0	0	6 (1.2)	11 (2.2)
Facelift	7 (2.5)	14 (5.0)	1 (0.5)	1 (0.5)	8 (1.6)	15 (3.0)
Cheek Implant	2 (0.7)	6 (2.2)	0	1 (0.5)	2 (0.4)	7 (1.4)
Otoplasty	1 (0.4)	2 (0.7)	2 (0.9)	1 (0.5)	3 (0.6)	3 (0.6)
Hair Transplantation	3 (1.1)	11 (3.9)	8 (3.7)	16 (7.5)	11 (2.2)	27 (5.5)
**Total**	**46 (16.5)**	**61 (21.9)**	**17 (7.9)**	**33 (15.4)**	**63 (12.8)**	**94 (19.1)**

**Table 3:** History of cosmetic surgery and the desire to perform it by level of mental and spiritual health and their dimensions

Variable	Total (%)	Have cosmetic surgery (Yes)		Desire to perform cosmetic surgery (Yes)	
		Number (%)	*p*‐value	Number (%)	*p*‐value
Mental health					
None	331 (67.1)	22 (6.6)		27 (8.2)	
Mild	95 (19.3)	16 (16.8)	*p* < 0.001	27 (28.4)	*p* < 0.001
Moderate or severe	67 (13.6)	25 (37.5)		40 (59.7)	
Somatic symptoms					
None	348 (70.6)	27 (7.8)		33 (9.5)	
Mild	113 (22.9)	24 (21.2)	*p* < 0.001	40 (35.4)	*p* < 0.001
Moderate or severe	32 (6.5)	12 (37.5)		21 (65.6)	
Anxiety symptoms					
None	335 (68.0)	23 (6.9)		28 (8.4)	
Mild	105 (21.3)	16 (15.2)	*p* < 0.001	28 (26.7)	*p* < 0.001
Moderate or Severe	53 (10.8)	24 (45.3)		38 (71.7)	
Social dysfunction					
None	343 (69.6)	25 (7.3)		30 (8.7)	
Mild	107 (21.7)	22 (20.6)	*p* < 0.001	34 (31.8)	*p* < 0.001
Moderate or severe	43 (8.7)	16 (37.2)		30 (69.8)	
Depression symptoms					
None	333 (67.5)	22 (6.6)		29 (8.7)	
Mild	100 (20.3)	18 (18.0)	*p* < 0.001	26 (26.0)	*p* < 0.001
Moderate or severe	60 (12.2)	23 (38.3)		39 (65.0)	
Spiritual health					
Low	85 (17.2)	9 (10.6)		12 (14.1)	
Moderate	385 (78.1)	53 (13.8)	0.34	80 (20.8)	0.16
High	23 (4.7)	1 (4.3)		2 (8.7)	
Religious health					
Low	85 (17.2)	9 (10.6)		12 (14.1)	
Moderate	367 (74.4)	50 (13.6)	0.63	77 (21.0)	0.18
High	41 (8.3)	4 (9.8)		5 (12.2)	
Existential health					
Low	104 (21.1)	12 (11.5)		14 (13.5)	
Moderate	354 (71.8)	49 (13.8)	0.36	76 (21.5)	0.09
High	35 (7.1)	2 (5.7)		4 (11.4)	
Total	493 (100)	63 (12.8)		94 (19.1)	

### 2.4 Ethics statements

This study was approved by the ethics committee of Kurdistan University of Medical Sciences with the code IR.MUK.REC.1400.181. Participation in the study was voluntary and written consent was obtained from the participants.

## 3 RESULTS

A total of 493 residents of Sanandaj participated in this study. Most of the respondents were female (56.6%) and had high school education (35.1%). The mean age of the subjects was 32.6 (standard deviation = 9.7) years and for those with the experience of cosmetic surgery was 33.4 (standard deviation = 9.9) years. The prevalence of cosmetic surgery was 12.8% in total (63 people) and 16.5% and 7.9% among men and women, respectively. The prevalence of inclination for performing cosmetic surgery was 19.1% in total and 21.9% and 15.4% among men and women, respectively. Other demographic and economic variables are listed in Table 1.

The most common type of performed cosmetic surgery was rhinoplasty with 5.5% (*n* = 27). Considering the gender, rhinoplasty with 7.2% (*n* = 20) and hair transplantation with 3.7% (*n* = 8) were the most common surgeries among women and men respectively. The most common desire to perform cosmetic surgery was for rhinoplasty with 6.9% (*n* = 34). Concerning the gender, the most common desire was for rhinoplasty with 7.9% (*n* = 22) and hair transplantation with 7.5% (*n* = 16) among women and men, respectively (Table 2).

According to Table 3, the prevalence of symptoms of mental disorders among people with the experience of cosmetic surgery and people willing to perform cosmetic surgery in total and by each dimension was significantly higher than the total number of subjects (*p* < 0.001). The prevalence of people with mild and moderate or severe mental disorders among subjects with a history of cosmetic surgery was 16.8% and 37.5%, respectively, and was 28.4% and 59.7% among those willing to have cosmetic surgery, respectively, which was significantly higher compared to the total number of subjects (*p* < 0.001). Also, there was a statistically significant difference in prevalence of somatic, anxiety, social dysfunction, and depression symptoms among people with a history of cosmetic surgery compared to people without the experience of performing cosmetic surgery, and among people willing to perform cosmetic surgery compared to those without a desire for such surgeries (*p* < 0.001) (Table 3).

Table 4 shows the determinants of having a history of performing cosmetic surgery and the desire to perform it in the adjusted model. The odds ratio of having an experience of cosmetic surgery were about 1.9 times more among women than men (odds ratio [OR] = 1.98; 95% confidence interval [CI]: 1.06–3.68). Also, it was three times more among people with mild symptoms (OR= 3.01; 95% CI: 1.06–3.68) and 7.6 times more among people with moderate or severe symptoms of mental disorder (OR = 7.59; 95% CI: 3.90–14.75) compared with asymptomatic people. The odds ratio of having cosmetic surgery were 4.4 times among people with mild symptoms of mental disorder (OR = 4.43; 95% CI: 2.42–8.11) and 15.8 times for those with moderate or severe symptoms (OR = 15.8; 95% CI: 8.3–29.9) in comparison to asymptomatic people (Table 4).

**Table 4:** Results of multivariate logistic regression of factors determining the history of having cosmetic surgery and the desire to perform it by study variables

Variable	Have cosmetic surgery		Desire to perform cosmetic surgery	
	Adjusted OR	CI	Adjusted OR	CI
Sex				
Female	1.98	1.06–3.68	1.02	0.55–1.92
Male	Ref		Ref	
Marital status				
Single	‐	‐	0.72	0.43–1.21
Married			Ref	
Employment status				
Employed	‐	‐	0.87	0.46–1.64
Unemployed			1.50	0.71–3.17
Housekeeper			Ref	
Household's income				
5 Million Toman>	Ref		‐	‐
5–10 Million Toman	1.22	0.68–2.21		
15 Million Toman<	0.51	0.17–1.57		
Mental health				
None	Ref		Ref	
Mild	3.01	1.50–6.04	4.43	2.42–8.11
Moderate or Severe	7.59	3.90–14.75	15.8	8.33–29.90
Spiritual health				
Low	‐	‐	Ref	
Moderate			1.44	0.70–2.99
High			0.51	0.09–291

Abbreviations: CI, confidence interval; OR, odds ratio.

Variables of Tables 1 and 3 with a *p*‐value < 0.2 were included in the model.

## 4 DISCUSSION

The aim of this study was to investigate the prevalence of cosmetic surgery and desire towards performing it as well as its relationship with mental and spiritual health in Sanandaj, Iran. The prevalence of cosmetic surgery and the desire to perform it were 12.8% and 19.1%, respectively. The most common type of cosmetic surgery was rhinoplasty with 5.5%. The prevalence of symptoms of mental disorders among people with cosmetic surgery and people willing to perform cosmetic surgery was significantly higher than all subjects.

In this study, the prevalence of having cosmetic surgery was 16.5% and 7.9% among women and men, respectively. Considering that Kurdistan province, with Sanandaj city as its center, is an economically poor and low‐income province, the high prevalence of cosmetic surgeries is worth considering. According to a study by Tavassoli et al. in Tehran (Iran), about 15.5% of women had undergone cosmetic surgery.^27^ According to another study in Tehran in 2015, which was conducted among people aged 15–60, 11% of the subjects had performed cosmetic surgery.^35^ In general, dissatisfaction with the appearance and the possibility of having cosmetic surgery are more among women than men.^36^ The high percentage of surgeries in this study is significant. In countries other than Iran, the ratio of women to men undergoing cosmetic surgery is about 9–1, but in studies conducted in Iran, this ratio is smaller, which indicates a greater desire of men to perform cosmetic surgery.^35^ In the present study, this ratio is about 2.

In our study, the prevalence of desire for performing cosmetic surgery was 19.1% in total and 21.9% and 15.4% among men and women, respectively; indicating a high desire for performing cosmetic surgery between both sexes. In recent years, cosmetic surgery in Iran, which is almost entirely provided by the private sector, has become very popular and is increasing. According to studies, the trend toward performing cosmetic surgery is increasing in the world ^16^, ^37^ and this trend can be attributed to medical advances, increased cosmetic services, increased number of cosmetic surgeons, reduced prices, and expanded media advertisements about cosmetic surgeries.^3^, ^10^ In the study of Soheylizad et al. in Iran, 26.7% of students had a desire for performing cosmetic surgery and this desire was twice as high among women.^28^ Women are more likely to have cosmetic surgery in other studies.^16^, ^38^

The most common type of cosmetic surgery performed by the subjects of the present study was rhinoplasty and the most inclination to perform cosmetic surgery was also for rhinoplasty. By gender, rhinoplasty for women and hair transplantation for men were the most common types of cosmetic surgeries. According to unofficial sources, Iran ranks first in the rate of rhinoplasty worldwide.^26^ In Alharethy's study, rhinoplasty was the most common type of surgery in Saudi Arabia.^39^ In the United States in 2020, top 5 cosmetic surgical procedures were rhinoplasty, eyelid surgery, facelift, liposuction, and breast augmentation, respectively.^5^ According to studies, there are differences in the preferences of different races for the type of cosmetic surgery.^40^

Another finding of the present study was that the prevalence of symptoms of mental disorders was significantly higher among people with a history of having cosmetic surgery and for people willing to perform cosmetic surgery. This finding is consistent with the results of other studies.^16^, ^41^, ^42^, ^43^ According to the study of Zahiroddin et al. in Iran, which was conducted as a case‐control study, there was no statistically significant difference between the mental health of candidates for performing rhinoplasty and the control group.^44^ In the study of Zojaji et al. in Iran, personality disorders were more in the group requesting rhinoplasty than the control group.^45^ In another study in Iran, mental disorders (depression, anxiety, aggression, morbidity, obsessive‐compulsive disorder, and sensitivity in relationships) were significantly higher in cosmetic surgery applicants than non‐applicants.^46^ However, in the study of Valikhani et al. in Iran, there was no significant difference between the two groups seeking cosmetic surgery and the control group in terms of depression, anxiety, stress, and vitality.^47^

According to the regression model in this study, the history of having cosmetic surgery had a significant relationship with female gender and having symptoms of mental disorder, while it had no relationship with education, income, marital status, employment status, and spiritual health. In the study of Bidkhori et al. in Tehran, the prevalence of cosmetic surgery was significantly related to gender and was 2.3 times higher among women than men. Also in the preceding study, age, income level, employment status, and level of education were influential factors on having cosmetic surgery.^35^

In the study of Salehahmadi et al. in Iran, social and psychological factors as well as demographic factors such as age, gender, and level of education had an impact on people's desire to perform cosmetic surgery.^48^ Results of other studies have indicated that more women than men undergo cosmetic surgery, which is consistent with the results of the present study.^39^, ^40^, ^49^ Studies in the United States and Saudi Arabia have reported a significant relationship between income levels and cosmetic surgery.^39^, ^40^ In various studies, having mental and personality disorders has been reported as a predictor of having cosmetic surgery and desire to perform it.^49^, ^50^

In our study, the desire towards having cosmetic surgery was only significantly associated with the mental health variable (having symptoms of mental disorder). In a study in Norway, symptoms of depression and anxiety were predictors of a desire to have cosmetic surgery.^49^ The results of the study of Soheylizad et al. in Iran are consistent with the findings of our study. In that study, there was no significant relationship between spiritual health and the desire for performing cosmetic surgery.^28^ According to a study by Swami et al. in Australia, being female and having a history of performing cosmetic surgery were among the factors influencing the desire to have cosmetic surgery.^50^

One of the limitations of this study was the collection of study data through self‐reporting. In addition, this study was conducted in the center of Kurdistan province and its results may not be generalizable to other parts of Iran, especially rural areas. Therefore, due to the limitations of the study, one should be careful in interpreting its results.

## 5 CONCLUSION

Both the prevalence and desire towards performing cosmetic surgery are high in Sanandaj and this needs the attention of health policymakers. Designing targeted interventions, such as performing mental health screening tests for cosmetic surgery candidates, with an emphasis on the findings of this study is recommended to reduce these practices.

## AUTHOR CONTRIBUTIONS

**Ahoora Ghorbani**: Conceptualization; methodology; writing – original draft. **Bakhtiar Piroozi**: Conceptualization; formal analysis; methodology; supervision; writing – original draft; writing – review & editing. **Hossein Safari**: Methodology; writing – original draft; writing – review & editing. **Azad Shokri**: Data curation; formal analysis; methodology; writing – review & editing. **Abbas Aqaei**: Formal analysis; writing – original draft. **Fayegh Yousefi**: Formal analysis. **Maziar Nikouei**: Data curation; writing – original draft. **Mahdi Rafieemovahhed**: Conceptualization; formal analysis; supervision; writing – original draft; writing – review & editing.

## CONFLICTS OF INTEREST

The authors declare no conflicts of interest.

## ETHICS STATEMENT

This study was approved by ethics committee of Kurdistan University of Medical Sciences with the code IR.MUK.REC.1400.81. Participation in the study was voluntary and written consent was obtained from the participants.

## TRANSPARENCY STATEMENT

The lead author Mahdi Rafieemovahhed affirms that this manuscript is an honest, accurate, and transparent account of the study being reported; that no important aspects of the study have been omitted; and that any discrepancies from the study as planned (and, if relevant, registered) have been explained.

## Data Availability

The data that support the findings of this study are available from the corresponding author (Mahdi Rafieemovahhed) upon reasonable request.
